# Supplementary figures and images for: IRF7-deficient MDCK cell based on CRISPR/Cas9 technology for enhancing influenza virus replication and improving vaccine production
Source: PeerJ. 2022 Sep 21;10:e13989. doi: 10.7717/peerj.13989 (PMC9508885; doi:10.7717/peerj.13989)

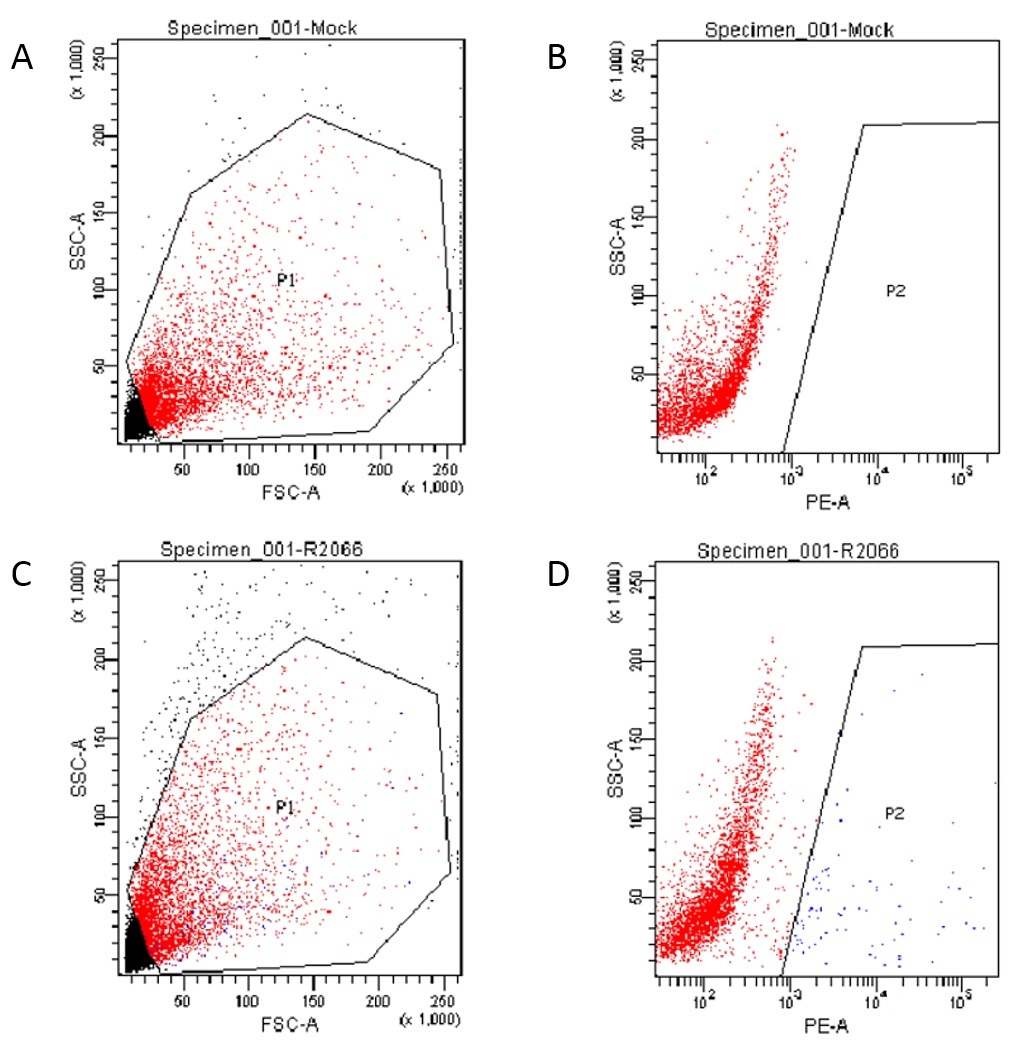

Supplement: Supplemental Information 1 — (A) and (B) represent negative control MDCK cells. (C) and (D) represent IRF7−/− MDCK cells. Cells with high OFP expression levels (P2 area) were isolated by fluorescence-activated cell sorting (FACS). [file peerj-10-13989-s001.jpg]

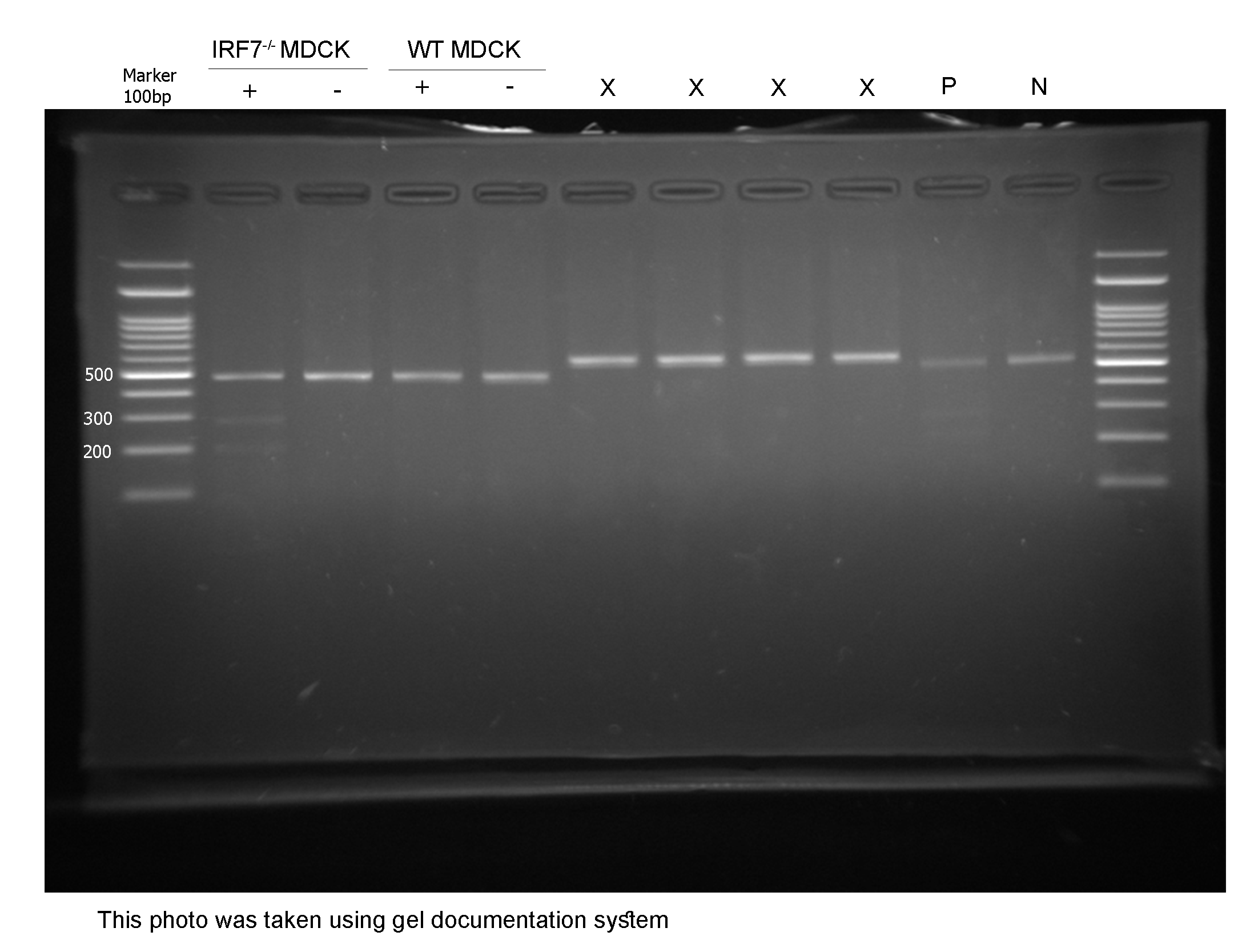

Supplement: Supplemental Information 4 — Raw image of agarose gel electrophoresis of cleavage detection displayed the parental band and cleavage bands from IRF7 −/− MDCK. [file peerj-10-13989-s004.png]

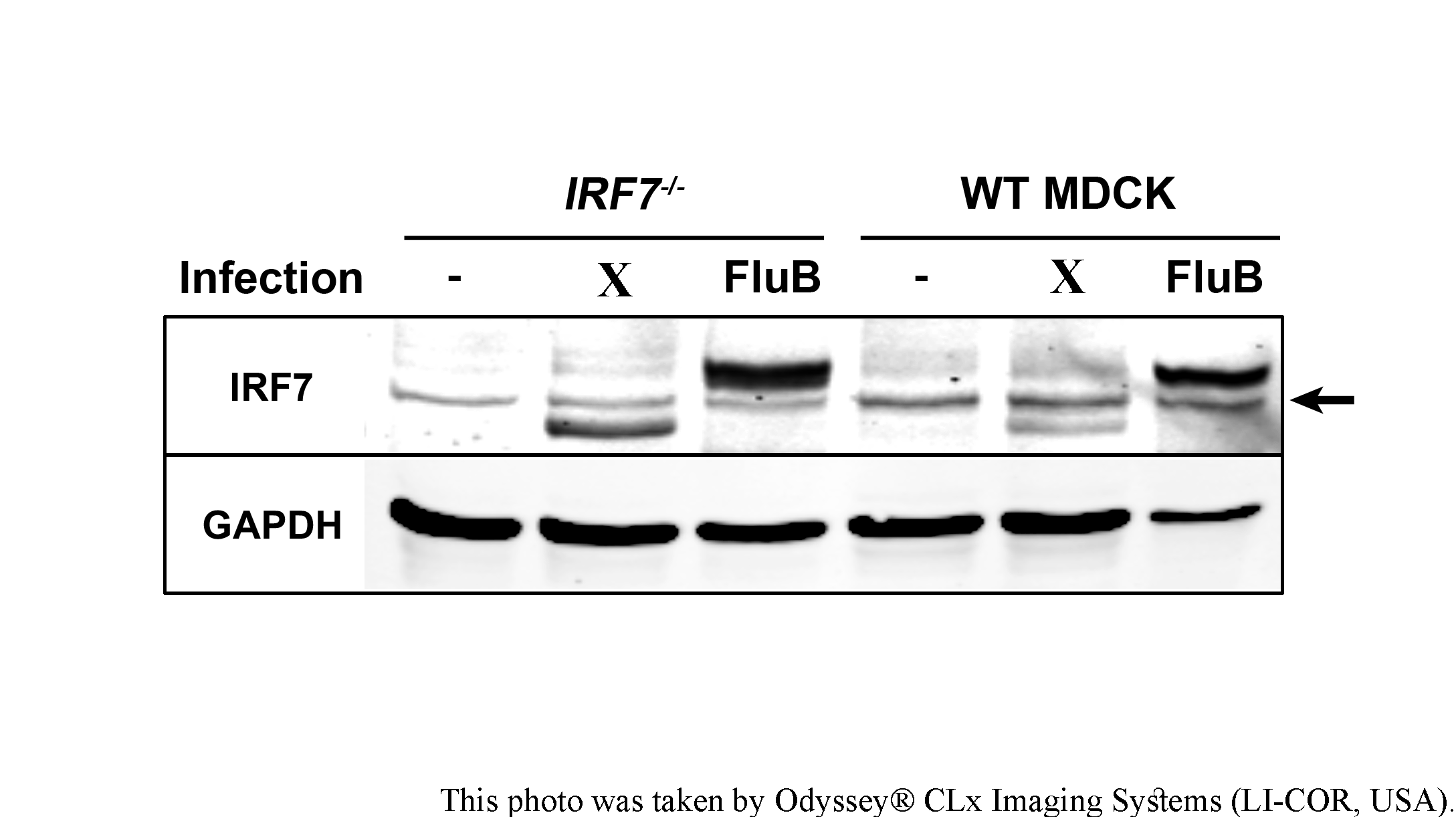

Supplement: Supplemental Information 5 [file peerj-10-13989-s005.png]
