# Supplementary material for: IRF7-deficient MDCK cell based on CRISPR/Cas9 technology for enhancing influenza virus replication and improving vaccine production
Source: PeerJ. 2022 Sep 21;10:e13989. doi: 10.7717/peerj.13989 (PMC9508885; doi:10.7717/peerj.13989)
Supplement: Supplemental Information 2 [file peerj-10-13989-s002.docx]

**Table S1. Summary result of influenza B (B/Massachusetts/2/2012) whole genome sequencing analysis.**

| progeny virus from | Total reads | Average length (bp) | Mapped read (%) | Average coverage |
| --- | --- | --- | --- | --- |
| WT MDCK | 544,298 | 108.46 | 87.97% | 3,714.60 |
| IRF7^-/-^ MDCK | 533,654 | 138.95 | 85.22% | 4,161.10 |
